# Measuring Gait Using a Ground Laser Range Sensor

**DOI:** 10.3390/s91109133

**Published:** 2009-11-17

**Authors:** Tomàs Pallejà, Mercè Teixidó, Marcel Tresanchez, Jordi Palacín

**Affiliations:** Department of Computer Science and Industrial Engineering, University of Lleida, Jaume II, 69, 25001 Lleida, Spain; E-Mails: tpalleja@diei.udl.cat (T.P.); mteixido@diei.udl.cat (M.T.); mtresanchez@diei.udl.cat (M.T.)

**Keywords:** gait measurement, laser range sensor, LIDAR

## Abstract

This paper describes a measurement system designed to register the displacement of the legs using a two-dimensional laser range sensor with a scanning plane parallel to the ground and extract gait parameters. In the proposed methodology, the position of the legs is estimated by fitting two circles with the laser points that define their contour and the gait parameters are extracted applying a step-line model to the estimated displacement of the legs to reduce uncertainty in the determination of the stand and swing phase of the gait. Results obtained in a range up to 8 m shows that the systematic error in the location of one static leg is lower than 10 mm with and standard deviation lower than 8 mm; this deviation increases to 11 mm in the case of a moving leg. The proposed measurement system has been applied to estimate the gait parameters of six volunteers in a preliminary walking experiment.

## 1. Introduction

The analysis of the human movement has applications in very different aspects of life sciences helping to understand how the human body works [1-3] and detect comparatively when something is wrong, for example to detect the effect in the gait of illnesses related with cerebral paralysis like Parkinson's disease [4,5] but other applications as human identification [6,7], gender classification [8], evaluation of the likelihood of falling in the elderly [9], *etc.* can be also developed.

Human locomotion and gait measurement are generally based on the tracking of external markers attached to the human body [6,7] but it can be also obtained using alternative techniques such as silhouette identification [7,10], attaching accelerometers and gyroscopes to the body [11], embedding sensors in the shoes [12], analyzing floor pressure profiles [13], *etc.*

In this work a ground laser range sensor is proposed as an alternative method for gait measurement. The use of laser range sensors for motion measurement or tracking was proposed recently by some authors [14-17] but, currently, remote two-dimensional laser range sensors are only used to get surrounding topographic scenario information for mobile robot navigation [16,17] and other volume correlated applications as the estimation of the foliage [18], specie [19], and microwave attenuation of some trees [20].

The main advantages of the use of a laser range sensor to register gait are that only one laser sensor placed with the scan plane parallel to the ground is needed to register the displacement of the legs. The laser range sensor allows medium range walking displacements comprising several strides. No external markers attached to the human body are needed. The system can be used in indoor and outdoor measurements, depending on the laser sensor. The system can be used in any ground surface without any visual references. The system does not require initial calibration or reference scales. The measurement system can be mounted everywhere in minutes. The main disadvantage of the basic measurement system is that only planar information of the position of the legs at a fixed height will be obtained, although as suggested by [14], multiple laser range sensors at different heights could be used to obtain additional body motion parameters.

This work is focused in the description of the measurement system as is outlined as follows: first, Section 2 describes the main characteristics of the selected laser range sensor and a full description of the steps and algorithms involved in the gait measurement system. Preliminary gait parameters of six volunteers are shown in Section 3 whilst the conclusions are presented in Section 4.

## 2. Measurement System

### 2.1. Laser Range Sensor

**Figure 1.**
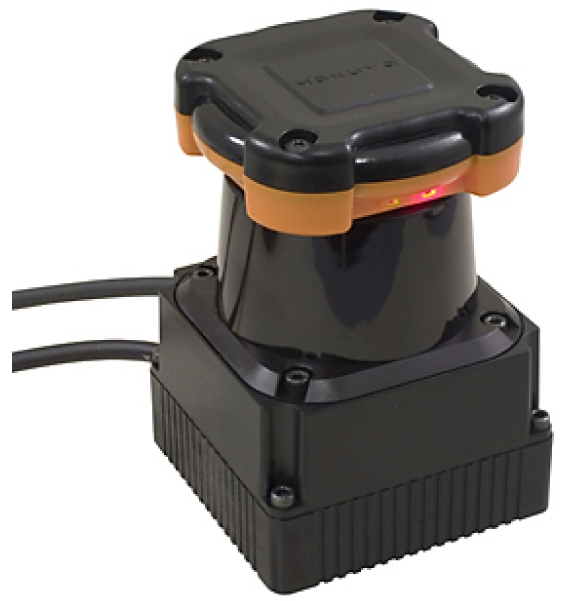
Hokuyo UTM-30LX.

The measurement system is based on a small prism laser range sensor attached to a portable computer for data storage. In this work, the Hokuyo UTM-30LX [21] a two-dimensional radial scanning laser range finder with an effective sensing range up to 30 meters in indoor and outdoor environments was selected (Figure 1). Table 1 resumes the main characteristics of the UTM unit: the laser requires a dedicated power source (12 V, 0.7 A), the scanning range is from −135° to +135° in steps of 0.25° (the 0° is in the front of the device) with a resolution of 1 mm, and one scan is completed in 0.025 s.

The UTM is attached to a portable computer using a USB 2.0 port. The USB connection emulates a RS232 serial port and only requires the installation (on the computer) of a proprietary USB driver to operate. The UTM is accessed using the standard commands of any serial port device and is controlled using a proprietary (open) protocol based on messages. The control program was implemented in a MatLab graphical user interface (GUI) taking advantage of its serial port functions and visualization capabilities. When started, the GUI searches for an UTM laser in all serial ports of the computer requesting device identification. The UTM automatically starts the two-dimensional scanning after power. When the serial communication port is open the laser can be requested to send the last scanned raw data to the computer in two different ways: one individual scan data or continuous scan mode (at 40 Hz). The raw data contains 1,081 distance-points expressed in millimeters and represented using 18 bits. Distances are divided in three bytes and coded in the ASCII range (values from 48 to 111). Distance-points are finally packed in a sequence of blocks of 64 bytes where 3,243 bytes represents a complete scan [21]. This unnecessary codification and packaging of the raw data is the main drawback of the UTM but, compared with other laser range sensors, an important advantage is the inclusion of an internal reference time (1 ms resolution) in each scan to avoid the effect of the delays originated in the USB serial communication with the computer. For the purpose of this paper any measurement data point *p_i_* obtained in the scan *j* is being defined by:

(1) $$p_{ij}=\left(t_{j},\alpha_{ij},d_{ij},x_{ij},y_{ij}\right)$$

where *t_j_* is the relative time at the start of the scan, *i* the index of the point in the scan, *α* the relative angular orientation of the beam, *d* the Euclidean distance measured and (*x,y*) the transformed Cartesian coordinates of the measured distance assuming a scan plane parallel to the ground with the laser placed at the origin and the Y axis defined by the angle 0° of the measurements. Then, the set of all laser points *S_j_* collected in the scan *j* is being defined by:

(2) $$S_{j}=\{p_{1j},p_{2j},\ldots ,p_{mj}\}$$

where *m* is the number of points of the scan, (*i.e.* 1,081 for the UTM).

### 2.2. Range measurement

**Figure 2.**
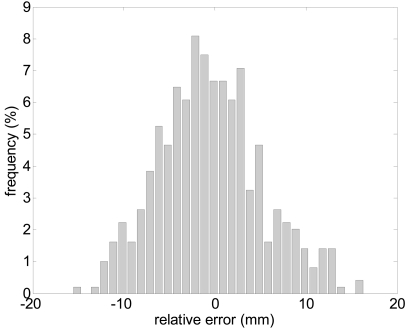
Histogram of the error in the distance measured: target at 8 m, angle 0°.

**Figure 3.**
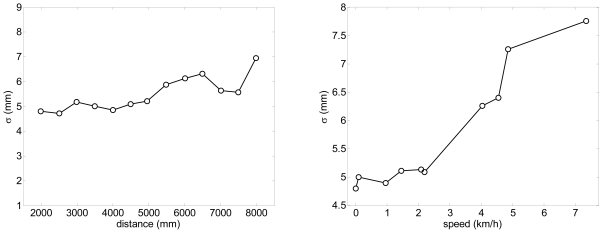
Left: evolution of the standard deviation relative to the distance (at angle 0°). Right: relationship between the standard deviation relative to the speed (at 5 m, angle 0°).

According the manufacturer, the accuracy expected in a single distance measured with the UTM is ±30 mm within a range up to 10 m and ±50 mm in the range from 10 to 30 m. Figure 2 shows the measured histogram of the error in the distance measured (at 5 m, angle 0°) relative to the average distance using a reflective target; the histogram has a Gaussian shape with a standard deviation of 5.6 mm. The maximum error in the measurement of the distance, ±16 mm, is under the manufacturer specifications. Figure 3-left shows the evolution of the standard deviation of the distance measured (at angle 0°) relative to the distance; the standard deviation slightly increases as the distance increases. Alternatively, Figure 3-right shows the relationship between the standard deviation of the distance measured and the speed of the target measured at 5 m. The standard deviation was obtained in a specific experiment with a plain target 100 mm wider (to simulate the width of a leg) to evaluate the effect of the time delay originated by the rotating profile of the consecutive points measured by the laser; the standard deviation only increases 3 mm at 7 km/h relative to the static case (0 km/h).

### 2.3. Estimating the Position of the Legs

**Figure 4.**
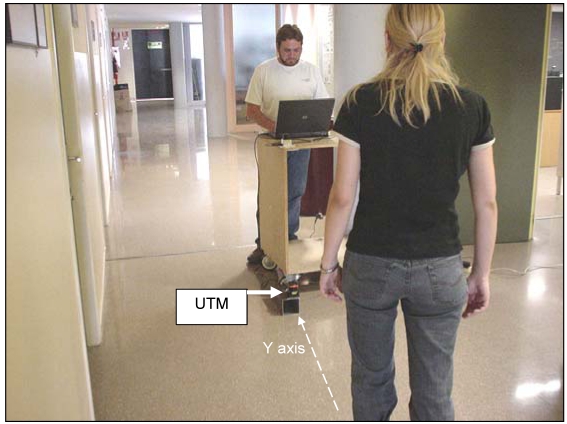
Example of the set of a typical gait measurement.

**Figure 5.**
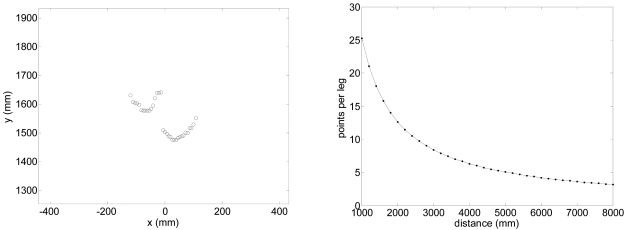
Left: typical scan data showing two legs of a walking volunteer. Right: estimation of the number of measurement points available in one leg relative to the distance.

Figure 4 shows an image of a typical gait measurement experiment. The UTM was placed at a height of 100 mm with the scanning plane parallel to the ground (approximately at the height of the ankle) to get the maximum information from the legs without detecting the shoes/feet during the walking. Figure 5-left shows an example of the raw data acquired with the measurement showing the legs of a walking volunteer. The contour of the leg is incomplete as the laser range system provides only one side information so an additional procedure will be needed to get an estimation of the center of the leg for the extraction of the gait parameters. Figure 5-right shows an analytical estimation of the number of strike laser points that define the contour of the leg relative to the distance; this number decreases exponentially as the distance increases due the radial scan measurement profile of the UTM.

The measurement points corresponding to the contour of the legs, ${\text{S}}_{j}^{L}$, can be extracted removing fixed points between consecutive scans or defining a valid rectangular measurement area. In this case, the creation of the subset is defined by:

(3) $${\text{S}}_{j}^{L}=\left\{p_{ij}\in S_{j}:X_{\text{MIN}}<x_{ij}\;X{<}_{\text{MAX}},Y_{\text{MIN}}<y_{ij}<Y_{\text{MAX}}\right\}$$

where *X_MIN_*, *X_MAX_*, *Y_MIN_*, *Y_MAX_* are the limits of the measurement area in Cartesian coordinates, in this work [−1000, 1000, 0, 8000].

In the case of a forward walking, the number of strike points corresponding to the left and right legs use to be the same with consecutive indexes. Then and the subset of each leg can be extracted with:

(4) $$\begin{array}{l} S_{j}^{LL}=\left\{p_{ij}\in {\text{S}}_{j}^{L}:i\leq cc_{j}\right\}\\ S_{j}^{RL}=\left\{p_{ij}\in {\text{S}}_{j}^{L}:i\geq cc_{j}\right\} \end{array}$$

where $S_{j}^{LL}$ and $S_{j}^{RL}$ are the subset of the left and right legs and *cc_j_* is the mean value of the index of the points included in the initial subset of the leg defined by:

(5) $$cc_{j}=\text{mean}\left\{i,p_{ij}\in {\text{S}}_{j}^{L}\right\}$$

The central point of an odd sequence is assigned to both legs and, if necessary, it will be later removed from the sequences as an outlier point.

Additionally, some measurement outliers can appear under certain circumstances in the border of the legs. These outliers are a direct consequence of the shape detection method used in laser range fingers that use to measure larger distances if the laser beam is partially reflected in the border of the target object. The outliers can be removed from $S_{j}^{LL}$ and $S_{j}^{RL}$ selecting a reference distance in the center of the initial sequence of the left and right leg, $m_{j}^{LL}$ and $m_{j}^{RL}$, computed with:

(6) $$\begin{array}{l} m_{j}^{LL}=\left\{d_{zj},p_{zj}\in S_{j}^{LL}:z=\text{median}\left\{i,p_{ij}\in S_{j}^{LL}\right\}\right\}\\ m_{j}^{RL}=\left\{d_{zj},p_{zj}\in S_{j}^{RL}:z=\text{median}\left\{i,p_{ij}\in S_{j}^{RL}\right\}\right\} \end{array}$$

and creating a final subset for each leg $S_{j}^{LO}$ and $S_{j}^{RO}$ removing the strike points that differ largely from the central reference distances:

(7) $$\begin{array}{l} S_{j}^{LO}=\left\{p_{ij}\in S_{j}^{LL}:\left|d_{ij}-m_{j}^{LL}\right|<k\right\}\\ S_{j}^{RO}=\left\{p_{ij}\in S_{j}^{RL}:\left|d_{ij}-m_{j}^{RL}\right|<k\right\} \end{array}$$

where *k* is a threshold value for outlier removal, 100 mm for the UTM.

Finally, the center of the legs can be estimated modeling each leg with a circle of fixed radius using a least mean square (LMS) fitting procedure [22]. To this end, let *R_ij_*(*u,v*) denote the distance between the point (*u,v*) and the data point *p_ij_* of the subset $S_{j}^{LO}$ or $S_{j}^{RO}$, adding a penalization factor, *w*, in case that (*u,v*) is closest than the data point to the origin, that is:

(8) $$R_{ij}(u,v)=\sqrt{{\left(x_{ij}-u\right)}^{2}+{\left(y_{ij}-v\right)}^{2}}+\left(\text{sign}(y_{ij}-v)+1\right)\cdot w$$

Let *μ*(*s,t*) a metric defined by:

(9) μ(s,t)=|s−t|

and let:

(10) $$G_{j}(u,v)=\sum_{i}{\left[\mu \{R_{ij}(u,v),r\}\right]}^{2}$$

that is the sum of squares of the distances from the data points to the circumference of the circle with centre (*u,v*) and fixed radius *r*.

A least squares estimator (*χ̂_j_,ψ̂_j_*) of the position of the center of the circle (*χ_j_,ψ_j_*) in the scan *j* is defined to be a value of (*u,v*) which globally minimizes *G_j_*(*u,v*), that is:

(11) $$G_{j}({\hat{\chi }}_{j},{\hat{\psi }}_{j})=\underset{u,v}{\min}G_{j}(u,v)$$

with initial values of (*u,v*) computed as the mean of the Cartesian location of the points of the subset $S_{j}^{LO}$ or $S_{j}^{RO}$, that is:

(12) $$\begin{array}{c} u=\text{mean}\{x_{ij}\}\\ v=\text{mean}\{y_{ij}\} \end{array}$$

At the end of the fitting procedure the position of the center of the leg *c_j_* in the scan *j* is defined by:

(13) $$c_{j}=(t_{j},{\hat{\chi }}_{j},{\hat{\psi }}_{j})$$

Then, the final set of the position of the leg *W* estimated for the *n* valid scans analyzed is defined by:

(14) $$W=\{c_{1},c_{2},\ldots ,c_{n}\}$$

In this work *w* = 1, *r* = 90 mm, and a minimum of three points per leg has fixed for the fitting procedure and thus the effective gait measurement range of the UTM is limited to 8 m (see Figure 5-right).

**Figure 6.**
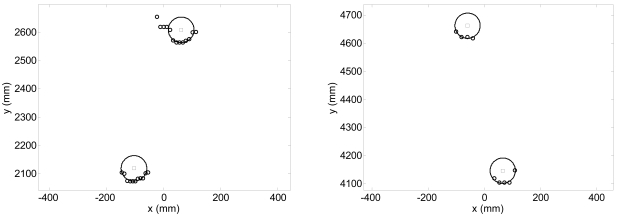
Example of fitting results for two legs at 2.4 (left), and 4.4 m (righ).

**Figure 7.**
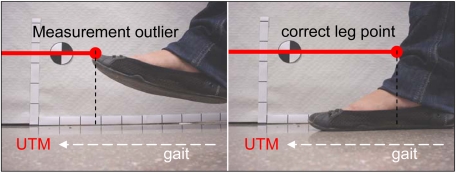
Example of an outlier point originated by the detection of the shoe.

Figure 6 shows the fitting results of the circles representing the legs in two different walking cases: Figure 6-left corresponds to a case where the walking volunteer is at 2.4 m with 10 points available per leg; in Figure 6-right the volunteer is at 4.4 m with 4 and 5 points per leg. Figure 7 shows a representation of additional outliers originated during the gait measurement by a shoe/foot if the height of the measurement plane is too low. These outliers denote a wrong gait measurement that must be avoided placing the laser at the adequate height by a trial and error procedure.

**Figure 8.**
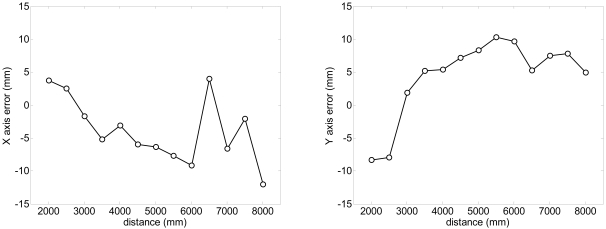
Evolution of the systematic absolute error of the averaged estimated position of the centre of the legs relative to the distance.

**Figure 9.**
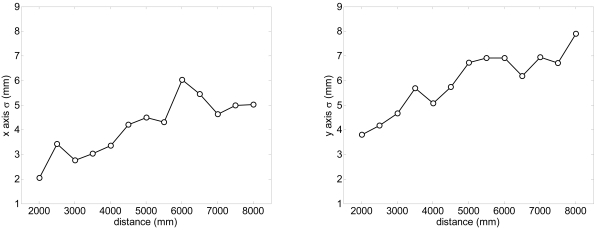
Evolution of the standard deviation of the estimated position of the centre of the legs relative to the distance.

A specific experiment with the legs placed statically at different distances was performed to estimate the systematic and random errors obtained in the estimation of the position of the center of the legs with the proposed fitting procedure evaluating with more than 100 scans per distance measured. Figure 8 shows the systematic absolute error relative to the distance; the error in the X (transversal) axis is lower than ±15 mm and lower to ±10 mm in the Y (longitudinal) axis in a range up to 8 m. Figure 9 shows the standard deviation obtained in the estimation of the position of the legs; in both axis the standard deviation increases as the distance increases but is lower than 10 mm in a range up to 8 m. Comparing the standard deviation shown in figures 3-right (obtaining analyzing only one distance point) and 9-left (obtained with all distance points of the contour of one leg), the fitting procedure is not able to reduce significantly the standard deviation of the estimated location of the center of the legs because of the circular and irregular shape of the target.

**Figure 10.**
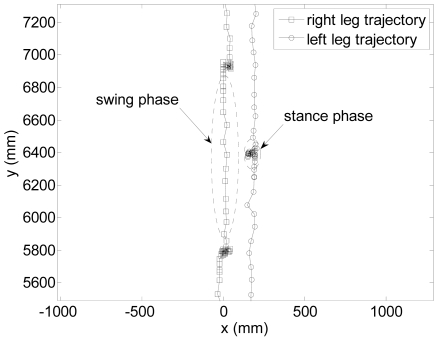
Example of trajectory of the legs estimated from the raw data.

Figure 10 shows the registered trajectory of the legs of one volunteer. The squares and the circles represents the estimated position of the center of the right and left legs during the walking experiment after the fitting procedure; in the swing phase the consecutive position of the legs are almost equally spaced between samples while in the stance phase the consecutive position of legs has an slightly oscillation in the transversal walking axis.

### 2.4. Step-Line Model of the Gait

**Figure 11.**
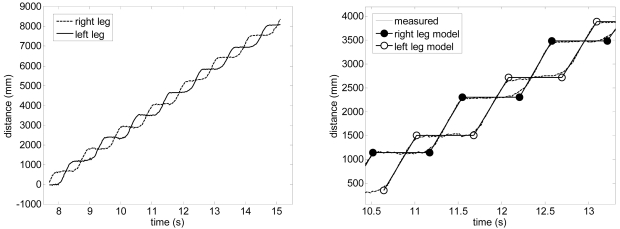
Left: leg displacement while walking. Right: detail of the step-line model.

Figure 11-left shows the relative distance run by both legs of one volunteer during the walking: in the swing phase the distance increases whereas in the stance phase the distance is almost constant (the foot is fixed in the ground). The evolution of each leg can be modeled with a sequence of step-lines to reduce the uncertainty in the determination of the phases: a plain line for the stance phase and a slopping line for the swing phase. To this end, let *P* represent the set of Boolean markers identifying if the position of the leg corresponds to a stance phase of the gait:

(15) $$P=\{s_{1},s_{2},\ldots ,s_{n}\}$$

where *s_i_* is computed with:

(16) $$\{\begin{array}{ccc} s_{i}=1 & \text{if} & \left|{\hat{\psi }}_{i}-{\hat{\psi }}_{i-1}\right|<\Delta \psi ,|{\hat{\psi }}_{i}-{\hat{\psi }}_{i+1}|<\Delta \psi \\ s_{i}=0 & \text{otherwise} \end{array}$$

where the typical threshold distance value Δ*ψ* is 100 mm for the UTM and the example cases analyzed in this work. Finally, any consecutive sequence of stance distance values larger than three points is averaged to get the Y coordinate of the plain line of the stance phase whereas the line of the swing phase is obtaining applying a linear regression to the remaining consecutive distance values between two stance phases (discarding two starting and ending points). The intersection of two adjacent stance and swing lines allows an accurate estimation of the position where the gait phase changes neglecting intermediate states and avoiding additional analytical computations. Figure 11-right shows the proposed step-line model (dark line) compared with the displacement estimated form the position of the center of the legs (dotted line): the circles depicts the estimated position of a change in the gait phases used to extract the parameters of the gait.

## 3. Results

**Figure 12.**
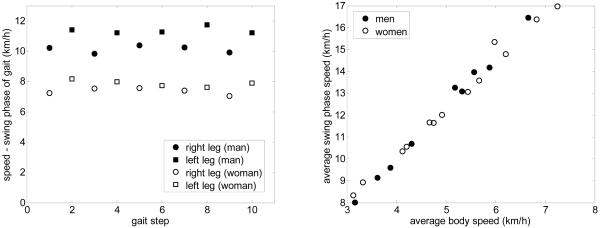
Left: Speed of the leg in the swing phase relative to the step. Right: Average swing phase speed relative to the average body speed.

**Figure 13.**
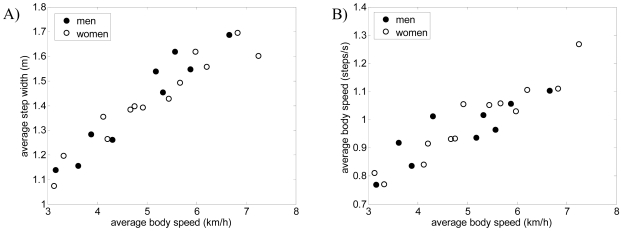

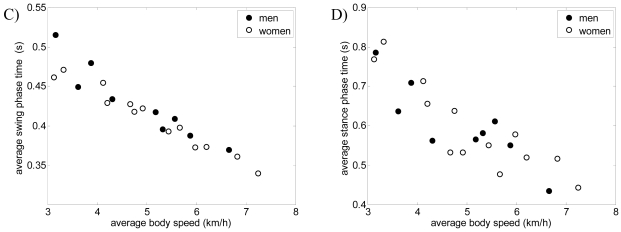
A) Average step width, B) average body speed in steps per second, C) average swing phase time, and D) average stance phase time relative to the average body speed.

As a preliminary gait measurement example, the walking displacement of six volunteers was measured in a large corridor: three men and three women of different height and ages. The walk started at 15 m and angle 0°, following a straight forward trajectory to the laser range sensor. Figures 12 and 13 show some gait parameters extracted from the walking measurements relative to the average body speed for three men and three women. Figure 12-left shows the speed of the legs during the swing phase of the gait for several steps in the case of one man and one woman walking at different forward speeds. The average speed of the swing phase of the gait for the man was 10.1 and 11.4 km/h for the right and left leg and 7.3 and 7.8 km/h for the right and left leg of the woman; the standard deviation was 0.2 km/h in all cases (refer to [23] for specific information of the normal range of gait parameters). Figure 12-right shows the average speed during the swing phase of the gait (averaging both legs) showing a very high correlation, 0.993, with the walking speed. Finally, Figure 13 shows some additional gait parameters obtained: the average step width (Figure 13-A), the average body speed in steps per second (Figure 13-B), and the swing (Figure 13-C) and stance (Figure 13-D) phase time.

## 4. Conclusions and Future Work

In this work a new gait measurement system based on a ground laser range sensor is proposed. The performances of the measurement system have been evaluated measuring gait parameters from a small group of volunteers.

The scan obtained with one two-dimensional laser range sensor allows the detection of the contour of the legs whose center is obtained fitting a circle with a specific LMS procedure. In a range up to 8 m the Y axis location of one static leg was located with a systematic error lower than 10 mm with an expected standard deviation lower than 8 mm, which increases to 11 mm in the case of a moving leg due to the radial scan profile of the laser range sensor.

The estimated displacement of the legs during the gait has been approximated with a step-line model to reduce the uncertainty in the determination of the change between the swing and stance phases of the gait, allowing precise estimation of several gait parameters as, for example: the relative trajectory and displacement of the legs, the distance and time spend in the swing and stand phase of the gait, the absolute speed of the legs, the average body trajectory, *etc.* As an example, the proposed measurement system has been applied to register the walking displacement of six volunteers in a large corridor to extract their gait parameters.

As a future work, the results obtained with the laser range sensor will be compared with other measurement systems used in applications where the analysis of the gait provides valuable medical or biomechanical information. Additionally, the measurement system will be also used to estimate the kinematics of the movement registered.

## Figures and Tables

**Table 1.** Hokuyo UTM-30LX Specifications.

**Parameter**	**Typical value**
Power source	12V ±10%
Current consumption	0.7A (rush current 1.0A)
Detection range	0.1 to approx. 60 m (<30 m guaranteed)
Laser wavelength	870 nm, Class 1
	Eye safe
Scan angle	270°
Scan time	0.025 s/scan (40.0 Hz)
Angular Resolution	0.25°
Interface	USB 2.0
	Serial COM
Weight	0.233 kg
Measurement error	0.1 to 10 m (±30 mm)
	10 to 30 m (±50 mm)
